# *In vitro* pharmacological evaluation of the safety and antibacterial efficacy of a Chilean honey hydrogel for veterinary wound applications

**DOI:** 10.3389/fphar.2026.1923388

**Published:** 2026-09-16

**Authors:** Andrea Julieta Müller-Sepúlveda, Liliam Monsalve, Carolina Campos-Estrada, Valeska Guevara-Torrejón, Ricardo Ceriani, Claudia Foerster

**Affiliations:** 1 Laboratorio de Farmacología, Instituto de Ciencias Agroalimentarias, Animales y Ambientales, Universidad de O’Higgins, San Fernando, Chile; 2 Laboratorio de Toxicología, Escuela de Química y Farmacia, Facultad de Farmacia, Universidad de Valparaíso, Valparaíso, Chile; 3 Centro de Investigación, Desarrollo e Innovación de Productos Bioactivos, Escuela de Química y Farmacia, Facultad de Farmacia, Universidad de Valparaíso, Valparaíso, Chile; 4 Laboratorio de Innovación Terapéutica y Diagnóstico Molecular, Escuela de Química y Farmacia, Facultad de Farmacia, Universidad de Valparaíso, Valparaíso, Chile; 5 Laboratorio de Inocuidad Alimentaria, Instituto de Ciencias Agroalimentarias, Animales y Ambientales, Universidad de O’Higgins, San Fernando, Chile

**Keywords:** Chilean honey, chitosan, fibroblast, hydrogel, *Quillaja saponaria* molina, *Staphylococcus pseudintermedius*

## Abstract

**Introduction:**

Honey-based biomaterials have gained increasing interest as topical strategies for wound-related applications because of their antimicrobial, antioxidant, and tissue-supportive properties. However, the direct use of raw honey is limited by its viscosity, variability, and lack of standardized delivery systems. This study aimed to perform a preliminary *in vitro* pharmacological evaluation of a Chilean honey hydrogel (CHH) formulated with honey, chitosan, and gelatin for potential veterinary wound-related applications.

**Methods:**

Eight Chilean honey samples with varying contributions of Quillay (*Quillaja saponaria* Molina) were physicochemical characterized before hydrogel preparation. CHH formulations containing different relative honey proportions were evaluated in NIH/3T3 murine fibroblasts using the resazurin reduction assay as an indirect indicator of cellular metabolic/reducing activity. Nitrite accumulation was assessed using the Griess method as an indirect marker of nitric oxide-mediated activation. Antibacterial activity against clinical isolates of *Staphylococcus pseudintermedius* was evaluated using agar diffusion and minimum inhibitory concentration assays, and the results were compared with those of raw honey and selected antibiotics.

**Results:**

All honey samples met basic physicochemical quality criteria. CHH formulations did not reduce fibroblast metabolic activity, whereas CHH-50% and CHH-100% significantly increased resazurin reduction compared with untreated control cells. Raw honey significantly increased nitrite accumulation, whereas CHH formulations did not increase nitrite levels under the experimental conditions tested. In agar diffusion assays, CHH produced larger and more homogeneous inhibition zones than raw honey against *S. pseudintermedius*, increasing the mean inhibition zone diameter from 10.98 ± 5.00 mm to 16.19 ± 1.82 mm. MIC assays further supported this effect, with CHH showing lower and less variable MIC values than raw honey. The median MIC decreased from 16.92 mg/mL for raw honey to 4.16 mg/mL for CHH, while MIC90 decreased from 104.17 to 10.42 mg/mL. As expected, amoxicillin–clavulanic acid and cefadroxil showed lower MIC values than CHH.

**Discussion:**

These findings support CHH as a biocompatible honey-derived formulation with improved antibacterial performance compared with raw honey under *in vitro* conditions. However, this study should be interpreted as a preliminary pharmacological evaluation rather than evidence of wound-healing efficacy. Further hydrogel characterization, mechanistic, bactericidal, canine skin cell, and *in vivo* studies are required to determine its translational relevance for veterinary wound management.

## 1 Introduction

Skin wounds and bacterial skin infections remain major therapeutic challenges in veterinary medicine, particularly in companion animals, where pyoderma is one of the most common dermatological conditions. *Staphylococcus pseudintermedius* is the main bacterial species associated with canine pyoderma and has become increasingly relevant because of its capacity to cause recurrent infections and develop resistance to commonly used antimicrobials (Kisseih et al., 2015; Loeffler and Lloyd, 2018; Priyantha, 2020; Lynch and Helbig, 2021). The emergence of methicillin-resistant and multidrug-resistant *S. pseudintermedius* strains has reinforced the need for alternative topical strategies capable of contributing to bacterial control while supporting tissue repair (Lynch and Helbig, 2021). In this context, bioactive biomaterials that combine antimicrobial activity, biocompatibility, and wound-supportive properties represent an important area of experimental and translational pharmacology.

Honey has been widely investigated as a bioactive natural matrix with antimicrobial, antioxidant, anti-inflammatory, and wound-healing properties (Minden-Birkenmaier and Bowlin, 2018; Nolan et al., 2019; Janda et al., 2020; Tashkandi, 2021). Its biological activity has been attributed to the combined action of high osmolarity, acidic pH, hydrogen peroxide generation, phenolic compounds, flavonoids, organic acids, and other non-peroxide factors, including bee-derived antimicrobial peptides (Kwakman et al., 2010; Alvarez-Suarez et al., 2016; Repellin et al., 2021; Ogwu and Izah, 2025). These mechanisms may contribute to bacterial inhibition, modulation of the wound microenvironment, reduction of oxidative stress, and stimulation of cellular processes involved in tissue repair (Molan and Rhodes, 2015; Minden-Birkenmaier and Bowlin, 2018; Martinello and Mutinelli, 2021). However, because honey is a chemically complex natural product, its pharmacological potential depends not only on its botanical and geographical origin but also on its physicochemical quality and degree of standardization (Dżugan et al., 2020).

The antioxidant properties of honey are particularly relevant for wound-related applications because the redox microenvironment influences inflammation, cellular viability, extracellular matrix remodeling, and tissue repair. Excessive reactive oxygen species can impair wound resolution, whereas controlled antioxidant activity may help modulate oxidative stress and support a more favorable microenvironment for healing (Alvarez-Suarez et al., 2016; Minden-Birkenmaier and Bowlin, 2018; Tashkandi, 2021). Honey-derived phenolic compounds, flavonoids, organic acids, enzymes, and other bioactive constituents have been associated with antioxidant capacity, and their abundance may vary according to botanical and geographical origin (Dżugan et al., 2020; Nolan et al., 2019). In Chilean honeys, botanical sources such as *Quillaja saponaria* Molina have been associated with distinctive phenolic profiles and antioxidant potential, supporting their interest as locally derived bioactive matrices for topical formulations (Bridi et al., 2017). Incorporating honey into a polymeric matrix, such as chitosan–gelatin, may improve handling and retention at the application site and help maintain the local availability of honey-derived antioxidant compounds. However, the extent to which the hydrogel matrix preserves, modifies, or controls the release of these antioxidant components requires specific physicochemical and release studies.

Despite its therapeutic potential, the direct application of raw honey has practical and pharmacotechnical limitations. Its high viscosity, variable composition, irregular spreading, and limited retention at the wound site can restrict reproducibility and clinical applicability (Minden-Birkenmaier and Bowlin, 2018; Holubová et al., 2021; Repellin et al., 2021). Therefore, incorporating honey into an appropriate delivery system is essential to improve handling, prolong contact with the wound surface, protect the injured tissue, and modulate the interaction between honey-derived bioactive compounds and the wound microenvironment. Hydrogel-based biomaterials are particularly attractive for wound applications because they can provide a moist environment, absorb exudate, support tissue interaction, and act as reservoirs for bioactive molecules (Elangwe et al., 2022; Liu et al., 2022; Jia et al., 2023). Recent studies and reviews have highlighted honey-loaded hydrogels as promising wound dressing platforms because they may combine the biological activity of honey with the structural and delivery advantages of polymeric matrices (Chopra et al., 2022; Yasin et al., 2023).

Among natural polymers, chitosan and gelatin are widely used in wound-related biomaterials because of their biocompatibility, film-forming capacity, biodegradability, and favorable interaction with cells involved in tissue repair (Chopra et al., 2022; Elangwe et al., 2022). Chitosan has also been associated with intrinsic antibacterial activity, mainly related to its cationic amino groups and their interaction with negatively charged bacterial surfaces, whereas gelatin can improve scaffold structure and provide cell-interactive motifs that may favor adhesion and tissue regeneration (Elangwe et al., 2022; Wang et al., 2023). Therefore, the combination of honey, chitosan, and gelatin may provide a simple and reproducible platform for the development of bioactive topical formulations with potential veterinary applications.

Chilean honeys, particularly those derived from central Chilean botanical sources such as *Quillaja saponaria* Molina, may have distinctive physicochemical and biological characteristics, including antioxidant and antimicrobial potential (Bridi et al., 2017). However, their incorporation into pharmacological biomaterials remains insufficiently explored. This is particularly important in wound care, especially in the veterinary field, where locally derived bioactive formulations could serve as complementary tools for topical care and the appropriate use of antimicrobials.

Despite increasing interest in honey-based wound dressings and hydrogels, limited evidence is available regarding the *in vitro* pharmacological safety and antibacterial efficacy of Chilean honey-based hydrogels against clinically relevant veterinary pathogens. Therefore, this study aimed to evaluate the safety and antibacterial efficacy of a simple Chilean honey hydrogel formulated with honey, chitosan, and gelatin. The formulation was assessed using murine fibroblasts as a cellular model relevant to tissue repair and clinical isolates of *S. pseudintermedius* as a bacterial model associated with canine skin infections. By integrating physicochemical standardization, cellular safety assessment, and antibacterial testing, this study provides experimental evidence to support the pharmacological evaluation of Chilean honey hydrogels as candidate topical biomaterials for veterinary wound-related applications.

## 2 Materials and methods

### 2.1 Physicochemical characterization of honey samples

Eight honey samples with different declared floral origins and variable contribution of Quillay (*Quillaja saponaria* Molina) were obtained from beekeepers in central Chile. Samples were stored in sterile amber containers at 15 °C and protected from light until analysis. Before hydrogel preparation, honey samples were characterized to confirm their physicochemical quality and to reduce variability associated with their natural origin. Diastase index (DI) was determined according to the harmonized methods of the European Honey Commission and expressed as Gothe units (Bogdanov et al., 1997). Hydroxymethylfurfural (HMF) concentration was measured by spectrophotometry, following (Pasias et al., 2017), and expressed in mg/kg. Soluble solids (SS) were determined using a refractometer and expressed as °Brix (Castillo Martínez et al., 2022), while color was classified using the Pfund scale and expressed as mm Pfund, and from white to dark amber. The pH and electrical conductivity (EC) were measured using a pH meter and a conductivity meter, respectively (Bogdanov, 2009). Antioxidant capacity (AC) was assessed using the DPPH radical scavenging assay and expressed as μg Trolox equivalents (TEAC)/g. Total phenolic content (TPC) was determined using the Folin–Ciocalteu method and expressed as μg gallic acid equivalents (GAE)/g (Santos et al., 2023). All determinations were performed in triplicate, and results were expressed as mean ± standard deviation.

### 2.2 Chilean honey hydrogels preparation

#### 2.2.1 CHH preparation

Chilean honey hydrogels (CHH) were prepared using a modified version of the method described by Wang et al. (2012). Briefly, 0.5 g of chitosan powder was dispersed in 19 mL of distilled water under continuous stirring, followed by the addition of 0.5 g of glacial acetic acid until complete chitosan dissolution. Separately, 20 g of honey was diluted with purified water at a 50:50 ratio (w/w), and 20 g of gelatin was dissolved in an equal weight of distilled water at 60 °C. The gelatin solution was then maintained under stirring at 40 °C, and the chitosan solution and diluted honey were gradually incorporated into the aqueous gelatin phase. Distilled water was added to obtain a final weight of 100 g. The resulting mixture was cast onto sterile watch glasses at a thickness of 2–4 mm and allowed to stand and cool at room temperature for 24 h to form the hydrogel. The final pH of the formulation was approximately 3.0. Hydrogels were freshly prepared before each experiment. Because preliminary assays showed that highly acidic chitosan-containing preparations affected fibroblast viability, the pH of formulations used in these cell culture assays was adjusted before cell exposure. The pH of the chitosan solution was adjusted to approximately 6.0 using triethanolamine before hydrogel preparation. Formulations with honey had a final pH of 5.0, and controls without honey of 6.0. The final pH of each formulation used in cell culture assays was measured before cell exposure. Chitosan, gelatin (Bovine skin, type B powder), glacial acetic acid, triethanolamine, and other analytical-grade reagents were purchased from Sigma-Aldrich (St. Louis, MO, USA).

In the present study, physicochemical characterization was performed on raw honey samples before hydrogel preparation to establish baseline quality and compositional parameters of the natural matrix. Specific active markers were not quantified in the final CHH formulation after incorporation into the chitosan–gelatin matrix. Therefore, post-formulation changes in phenolic content, antioxidant capacity, peroxide-related activity, non-peroxide factors, and release behavior were not determined in this work.

#### 2.2.2 Assessment of visible microbial contamination

Before *in vitro* assays, aliquots of each hydrogel formulation were incubated at 37 °C in a humidified atmosphere containing 5% CO_2_ for 48 h to assess visible microbial contamination. Samples were inspected microscopically every 24 h. No visible bacterial or fungal contamination was observed under these conditions.

### 2.3 Determination of the *in vitro* effect of CHH on fibroblasts

#### 2.3.1 Concentrations and controls

To evaluate the *in vitro* effect of CHH on fibroblasts, control formulations containing chitosan, honey, gelatin, and their respective combinations were prepared (Table 1). All preparations were made using distilled water. Three CHH formulations were prepared according to the relative honey content incorporated into the gelatin–chitosan matrix: CHH-10%, CHH-50%, and CHH-100%. These formulations corresponded to honey:gelatin:chitosan proportions of 0.2:1:0.025, 1:1:0.025, and 2:1:0.025, respectively.

**TABLE 1:** Compounds and controls used to determine the *in vitro* effect of CHH on fibroblast cells.

Compound(s)	Abbreviation	Concentration or proportion
Chitosan	Q	0.5% chitosan
Honey	M	20% honey
Gelatin	G	10% gelatin
Chitosan + gelatin	Q + G	Q-G 2 : 0.025
Chitosan + honey	Q + M	Q-M 0.025 : 2
Gelatin + honey	M + G	M-G 1 : 2
CHH-10%	CHH-10%	M-G-Q 0.2 : 1: 0.025
CHH-50%	CHH-50%	M-G-Q 1 : 1: 0.025
CHH-100%	CHH-100%	M-G-Q 2 : 1: 0.025

Before cell culture assays, preliminary dilution tests were performed to assess the behavior of the hydrogel matrices in culture medium. A small portion of each hydrogel was added to 5 mL of high-glucose DMEM supplemented with L-glutamine, 10% heat-inactivated fetal bovine serum, 1% penicillin/streptomycin, and 1% Plasmocin. After 5 min, matrix swelling and partial solidification at room temperature were observed. Based on these observations, hydrogel matrices were freshly prepared before each cell culture experiment.

#### 2.3.2 Fibroblast culture conditions

Murine NIH/3T3 fibroblasts were obtained from the American Type Culture Collection (ATCC, Manassas, VA, USA). Cells were maintained in Dulbecco’s Modified Eagle’s Medium (DMEM) supplemented with 5 U/mL penicillin–streptomycin and 10% bovine calf serum. Cells were cultured at 37 °C in a humidified atmosphere containing 5% CO_2_, as previously described (Premarathna et al., 2020).

NIH/3T3 fibroblasts were selected as a standardized preliminary *in vitro* model for assessing cytocompatibility and wound-relevant cellular responses, given the central role of fibroblasts in extracellular matrix deposition, tissue remodeling, and wound repair. However, this cell line was used as an initial screening model and does not replace canine dermal cell models.

#### 2.3.3 Cell viability assay

Cell metabolic activity, used as an indirect indicator of cell viability, was assessed using the resazurin reduction assay (Kamiloglu et al., 2020). NIH/3T3 fibroblasts were seeded into 96-well flat-bottom culture plates at a density of 2 × 10^4^ cells/well in 100 μL of culture medium and incubated for 24 h at 37 °C in a humidified atmosphere containing 5% CO_2_. After this period, 100 μL of each experimental condition described in Table 1 was added to the corresponding wells, and plates were incubated for an additional 24 h.

After incubation, the supernatant was removed and stored in pre-labeled Eppendorf tubes at −80 °C for subsequent analyses. Then, 150 μL of resazurin solution was added to each well at a final concentration of 44 μM, and plates were incubated for 4 h at 37 °C in the dark. Fluorescence was measured at 560/590 nm using a microplate reader. Fluorescence values were normalized to untreated control cells, which were considered 100%. Results correspond to the mean ± standard deviation of three independent experiments and are expressed as a percentage of cell viability relative to untreated control cells.

#### 2.3.4 Assessment of nitrite production

Nitric oxide production was indirectly assessed by measuring nitrite accumulation in the culture supernatant using the Griess Reagent Kit (Thermo Fisher Scientific), according to the manufacturer’s instructions. NIH/3T3 fibroblasts were seeded into 96-well flat-bottom culture plates at a density of 3 × 10^4^ cells/well in 100 μL of culture medium and incubated for 24 h at 37 °C in a humidified atmosphere containing 5% CO_2_.

After this period, cells were stimulated with LPS (1 μg/mL) and exposed to 100 μL of each experimental condition. After incubation, 150 μL of culture supernatant was collected and centrifuged at 2000 rpm for 5 min. Subsequently, 150 μL of supernatant was transferred to a 96-well plate, followed by the addition of 130 μL of distilled water and 20 μL of Griess reagent for each treatment. Plates were incubated at room temperature for 30 min, and absorbance was measured at 548 nm using a microplate reader.

Nitrite concentrations were calculated from a sodium nitrite standard curve prepared in culture medium (1–100 μM). Results correspond to the mean ± standard deviation of four independent experiments and are expressed as a percentage relative to LPS-stimulated control cells.

### 2.4 Determination of the *in vitro* antibacterial effect of CHH against *Staphylococcus pseudintermedius*

#### 2.4.1 Bacterial culture

For antibacterial assays, a modified CHH formulation was used to allow diffusion through Mueller–Hinton agar. Preliminary assays showed that the gelatin concentration used in the cell culture formulation limited the diffusion of hydrogel components in agar. Therefore, the gelatin content was reduced, resulting in a honey:gelatin:chitosan ratio of 5:1:0.05. This modified formulation was used exclusively for agar diffusion assays. This methodological adjustment was intended to evaluate antibacterial activity under agar diffusion conditions and should be considered when comparing these results with those obtained using the cell culture formulation.

Fourteen clinical isolates of *S. pseudintermedius* were obtained from dogs examined at a Veterinary Clinical Hospital. Samples were collected from the perianal area and nasal mucosa and cultured on mannitol salt agar at 37 °C for 24 h. Presumptive *Staphylococcus* colonies were selected based on colony morphology, Gram staining, catalase activity, and coagulase testing. Species-level identification was confirmed by PCR, as previously described (Abusleme et al., 2022). Isolates were stored at −20 °C in peptone water until analysis. Before antibacterial testing, isolates were subcultured on Mueller–Hinton agar and incubated at 37 °C for 24 h under aerobic conditions. *Staphylococcus aureus* ATCC 43300 was used as a reference control strain.

#### 2.4.2 Agar diffusion assay

The antibacterial activity of raw honey and CHH against *S. pseudintermedius* was evaluated using an agar well diffusion assay. Antimicrobial susceptibility to commercial antibiotics was assessed using the Kirby–Bauer disk diffusion method according to Clinical and Laboratory Standards Institute guidelines (Wayne, 2015). Bacterial suspensions were prepared from overnight cultures and adjusted to 0.5 McFarland. Using a sterile cotton swab, bacterial suspensions were uniformly inoculated onto Mueller–Hinton agar plates. Freshly prepared CHH and raw honey were maintained at 40 °C before application. Wells of 8 mm diameter and 5 mm depth were created in the agar, and 300 mg of raw honey or CHH was added to each well. Blank wells/discs were used as negative controls.

The antibacterial treatments included raw honey (100%) and the modified CHH formulation with a honey:gelatin:chitosan ratio of 5:1:0.05. Commercial antibiotic disks commonly used in the treatment of canine pyoderma were also evaluated, including amoxicillin–clavulanic acid (30 μg), cefadroxil (30 μg), clindamycin (2 μg), and doxycycline (30 μg). Additional antibiotics were included to characterize the antimicrobial susceptibility profile of the isolates: cefpodoxime (10 μg), cefovecin (30 μg), chloramphenicol (30 μg), minocycline (30 μg), lincomycin (2 μg), and sulfamethoxazole–trimethoprim (25 μg) (Thermo Fisher Scientific, Basingstoke, UK).

Plates were incubated at 37 °C for 24 h under aerobic conditions. After incubation, inhibition zone diameters were measured in millimeters. Each isolate was tested in duplicate for each treatment.

Since no standardized interpretive criteria are available for honey or honey-based hydrogels against *S. pseudintermedius*, inhibition zone diameters were primarily analyzed as continuous variables. For exploratory purposes, a 17-mm threshold was used as a reference value based on CLSI oxacillin disk diffusion interpretive criteria previously proposed for *S. pseudintermedius*. This threshold was applied only to compare the relative categorical shift between raw honey and CHH formulations and should not be interpreted as a validated clinical susceptibility breakpoint for honey- or hydrogel-based treatments.

#### 2.4.3 Minimum inhibitory concentration assay

The minimum inhibitory concentration (MIC) was determined using a broth microdilution assay according to CLSI recommendations, with modifications required for honey and hydrogel matrices. Serial two-fold dilutions of raw honey and CHH were prepared in Mueller–Hinton broth to obtain final concentrations ranging from 7.8 to 500.00 mg/mL. For CHH, concentrations were expressed according to the honey-equivalent content released from the formulation, ranging from 3.12 to 16.67 mg/mL. Each well contained 100 μL of test solution and 50 μL of bacterial suspension, reaching a final inoculum of 1–5 × 10^5^ CFU/well. Plates were incubated at 37 °C for 20 h under aerobic conditions. The inhibitory effect was compared with the antibiotics amoxicillin–clavulanic acid and cefadroxil, which were used at concentrations ranging from 0.08 to 5.00 mg/L. MIC was defined as the lowest concentration at which no visible bacterial growth was observed. Final MIC values were calculated from triplicate determinations and used for descriptive analysis.

### 2.5 Statistical analysis

For fibroblast assays, normally distributed data were analyzed by one-way ANOVA using GraphPad Prism 8.0 software. When appropriate, multiple comparisons against the corresponding control group were performed using Dunnett’s *post hoc* test. Results from cell-based assays are expressed as mean ± standard deviation from independent experiments.

For antibacterial assays, inhibition zone diameters obtained from paired raw honey and CHH formulations were compared using the Wilcoxon signed-rank test. Categorical shifts based on the exploratory 17-mm reference threshold were analyzed using McNemar’s test. Comparisons between CHH and commercial antibiotics were performed using the Mann–Whitney U test. MIC values were analyzed descriptively and, when paired comparisons were possible, raw honey and CHH MIC distributions were compared using the Wilcoxon signed-rank test. For this analysis, the median MIC values for raw honey were summarized by isolate before being compared with the corresponding MIC value for CHH.

Correlations between honey physicochemical parameters and antibacterial activity were assessed using Pearson’s correlation coefficient. Because only eight honey samples were included, correlation analyses were considered exploratory and hypothesis-generating. Correlation results were not used to establish causality or definitive predictors of antibacterial activity. Multiple comparisons in cell-based assays were addressed using Dunnett’s *post hoc* test; antibacterial comparisons were interpreted according to pre-specified pairwise hypotheses, and exploratory correlation analyses were interpreted cautiously due to the limited sample size. Statistical significance was set at *p* < 0.05. Antibacterial and correlation analyses were performed using R software (R Core Team) via RStudio version 2025.09.1 + 401.

## 3 Results

### 3.1 Physicochemical characterization of honey samples

The physicochemical characterization of the eight honey samples showed values consistent with basic quality criteria for honey (Table 2). All samples exhibited diastase index (DI) values above 8 Gothe units and hydroxymethylfurfural (HMF) concentrations below 40 mg/kg. Soluble solids, expressed as °Brix, were above 75°Brix in all samples. Honey color varied according to floral origin, ranging from white to dark amber on the Pfund scale. The pH values were within the expected acidic range for honey, ranging from 3.77 to 4.51, while electrical conductivity (EC) values were below 800 μS/cm in all samples. Regarding bioactive parameters, antioxidant capacity (AC) values were above 200 µg TEAC/g, and total phenolic content (TPC) values were above 270 µg GAE/g. Overall, these results indicate that the honey samples met basic physicochemical quality criteria and presented measurable antioxidant and phenolic profiles suitable for subsequent hydrogel preparation.

**TABLE 2:** Physicochemical parameters of honey samples.

Parameter/Honey sample	M1	M2	M3	M4	M5	M6	M7	M8
Floral origin	Multi-floral	Mono-floral	Multi-floral	Mono-floral	Multi-floral	Multi-floral	Multi-floral	Mono-floral
% Quillay	42%	82%	49%	91%	19%	19%	10%	52%
DI (gothe units)	21.08	32.40	9.68	37.43	25.20	19.75	16.76	34.73
HMF (mg/kg)	3.70	2.20	24.40	9.80	4.64	4.20	7.20	1.50
Soluble solids (°Brix)	84.40	83.40	81.40	77.60	84.30	80.60	83.00	83.80
Color (mm Pfund)	41.93	217.60	60.68	73.68	83.61	33.76	98.38	118.52
Pfund color classification	Extra-light amber	Dark amber	Light amber	Light amber	Light amber	White	Amber	Dark amber
pH	4.30	4.22	4.19	4.38	3.77	4.37	4.51	4.31
EC (µS/cm)	423.00	572.00	374.00	719.00	451.00	599.00	288.00	375.40
AC (µg TEAC/g)	263.01	259.35	348.37	250.81	405.75	229.47	261.38	409.86
TPC (µg GAE/g)	274.77	312.45	273.12	276.18	460.00	318.87	283.27	619.34

M1 - M8 correspond to the eight honey samples. DI, diastase index; HMF, hydroxymethylfurfural; EC, electrical conductivity; AC, antioxidant capacity; TPC, total phenolic content. In this study, monofloral honey is defined as honey that consists of more than 50% of a single floral source.

Although the honey samples used for CHH preparation showed measurable antioxidant capacity and total phenolic content, the antioxidant behavior of the final hydrogel formulation was not directly assessed in this study. Therefore, the contribution of honey-derived antioxidant compounds to the biological response observed in fibroblasts remains hypothetical.

### 3.2 Determination of the *in vitro* effect of CHH on fibroblasts

#### 3.2.1 Effect of CHH on the metabolic activity of murine fibroblasts

The resazurin reduction assay showed that CHH formulations did not reduce NIH/3T3 fibroblast metabolic activity under the experimental conditions evaluated (Figure 1). Among all treatments, only CHH-50% and CHH-100% induced a significant increase in metabolic activity compared with untreated control cells, reaching values approximately 25%–30% higher than control levels (p < 0.0021). These results indicate that the hydrogel matrix containing higher relative honey content was not cytotoxic under the experimental conditions tested. The increased resazurin reduction observed with CHH-50% and CHH-100% should be interpreted as increased cellular metabolic/reducing activity rather than direct evidence of increased cell number or fibroblast proliferation.

**FIGURE 1:**
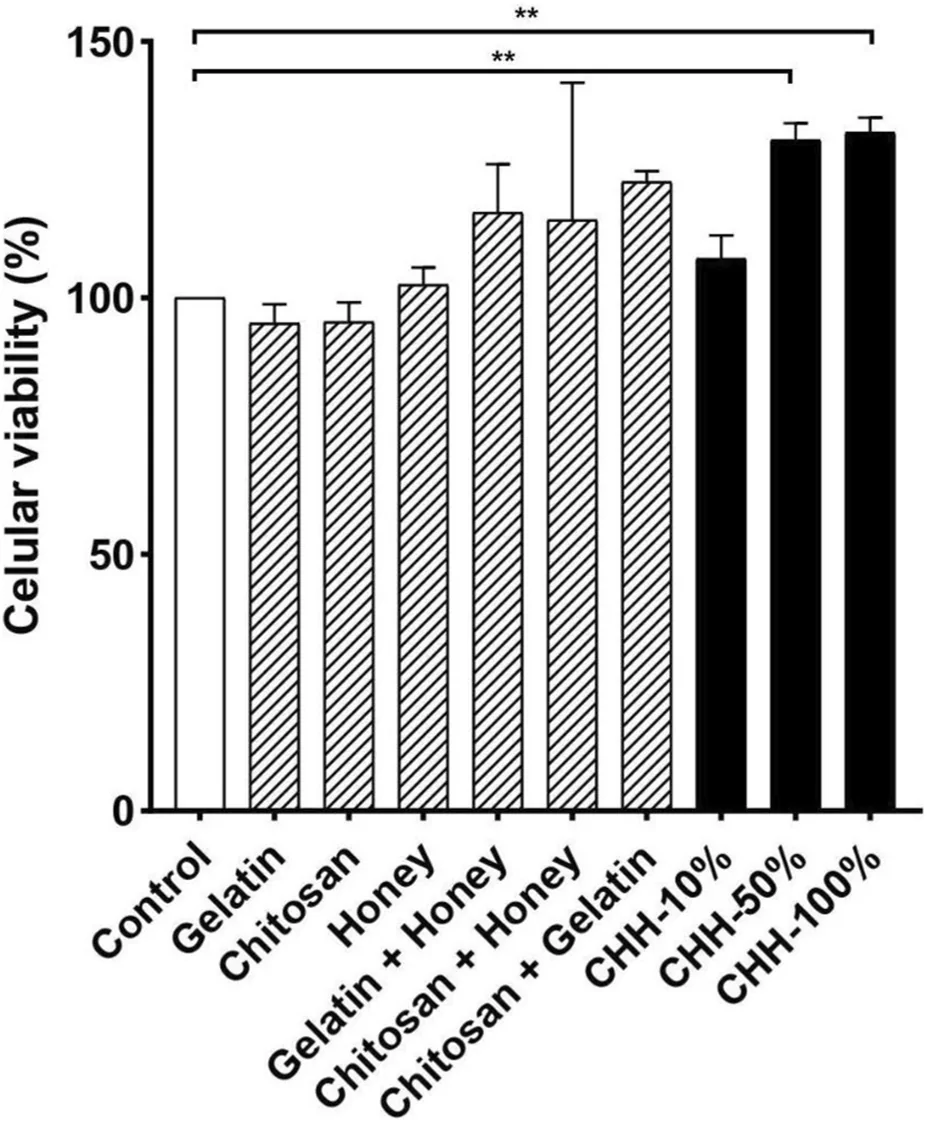
Assessment of cell metabolic activity using the resazurin reduction assay in NIH/3T3 fibroblasts incubated under different experimental conditions. Data represent the mean ± standard deviation from three independent experiments. Statistical analysis was performed using one-way ANOVA. Statistical significance was defined as **p < 0.0021 compared with untreated control cells.

The individual components and binary combinations did not significantly reduce metabolic activity compared with control cells. Although some treatments containing honey presented a tendency to increase metabolic activity, these changes did not reach statistical significance. These findings indicate that CHH formulations, particularly CHH-50% and CHH-100%, were biocompatible with NIH/3T3 fibroblasts and enhanced cellular metabolic activity under the conditions tested.

#### 3.2.2 Effect of CHH on nitrite production in murine fibroblasts

Nitric oxide production was indirectly assessed by measuring nitrite accumulation in the culture supernatant using the Griess method (Figure 2). Among the experimental conditions evaluated, only raw honey significantly increased nitrite levels compared with LPS-stimulated control cells. In contrast, none of the CHH formulations increased nitrite production relative to the LPS-stimulated control.

**FIGURE 2:**
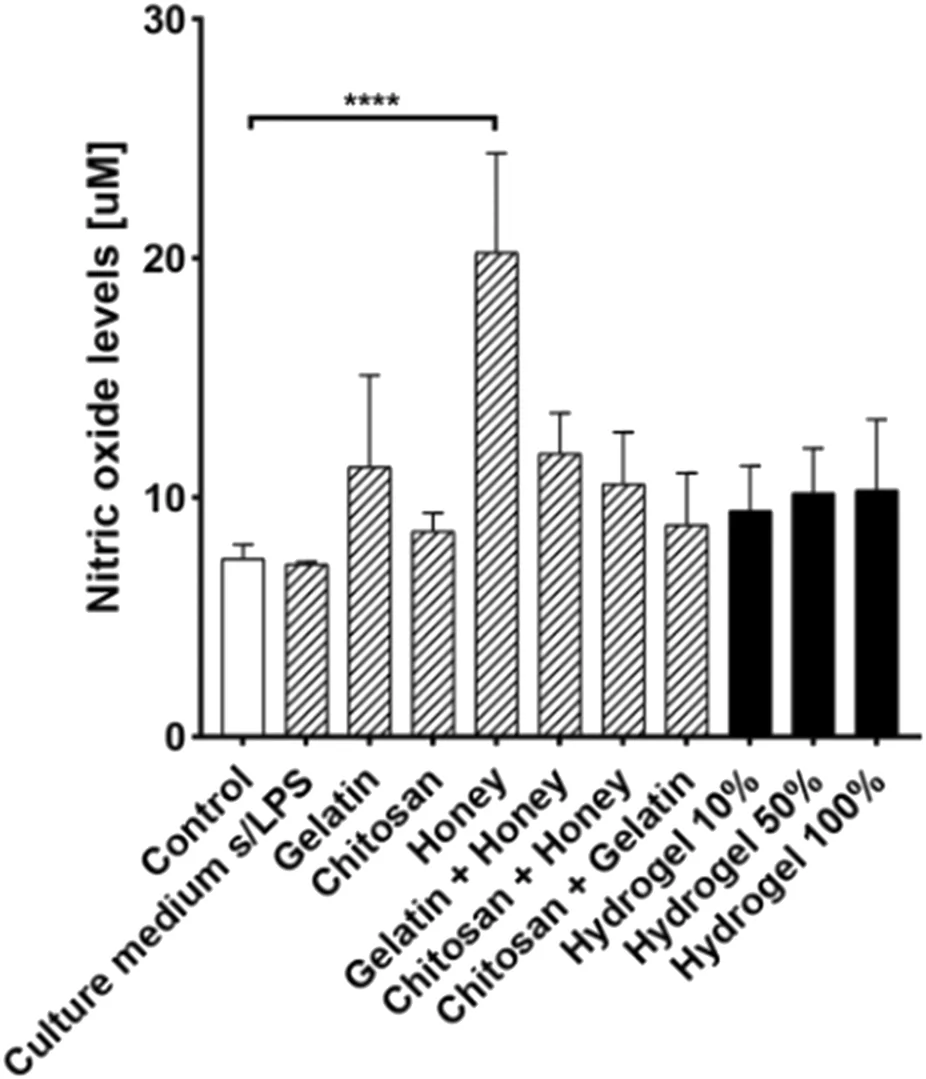
Assessment of nitrite production using the Griess method in NIH/3T3 fibroblasts incubated under different experimental conditions. Data represent the mean ± standard deviation from four independent experiments. Statistical analysis was performed using one-way ANOVA. Statistical significance was defined as ****p < 0.0001 compared with LPS-stimulated control cells.

These results suggest that, under the experimental conditions tested, CHH formulations did not induce nitric oxide-mediated inflammatory activation in NIH/3T3 fibroblasts. Moreover, incorporation of honey into the gelatin–chitosan hydrogel matrix appeared to prevent the increase in nitrite levels observed with raw honey alone.

### 3.3 Determination of the *in vitro* antibacterial effect of CHH against *Staphylococcus pseudintermedius*

The agar diffusion assay showed that raw honey samples produced variable inhibition against the 14 clinical isolates of *S. pseudintermedius* (Table 3). Inhibition zone diameters for raw honey ranged from 0 to 26 mm, with a mean value of 10.98 ± 5.00 mm. In contrast, CHH formulations produced inhibition zones ranging from 12 to 20 mm, with a mean value of 16.19 ± 1.82 mm. Incorporation of honey into the hydrogel matrix significantly increased antibacterial activity compared with raw honey, as supported by the Wilcoxon signed-rank test (*p* < 0.001).

**TABLE 3:** Inhibition zone diameters of raw honey, CHH, and commercial antibiotics against clinical isolates of *Staphylococcus pseudintermedius*.

Treatment	Observations	Range	Mean ± SD
Raw honey	112	0–26 mm	10.98 ± 5.00 mm
CHH	112	12–20 mm	16.19 ± 1.82 mm
Commercial antibiotics, overall panel	140	0–43 mm	25.64 ± 10.65 mm

Since no standardized interpretive criteria are available for honey or honey-based hydrogels against *S. pseudintermedius*, inhibition zone diameters were primarily analyzed as continuous variables. As a secondary exploratory analysis, a 17-mm reference threshold was applied. Using this threshold, 45 CHH observations reached or exceeded 17 mm, compared with only two observations for raw honey. This categorical shift was supported by McNemar’s test (*p* < 0.001). However, this threshold should be interpreted only as an exploratory comparator and not as a validated clinical susceptibility breakpoint for honey- or hydrogel-based treatments.

The commercial antibiotic panel displayed greater mean inhibition zone diameters than CHH, with values ranging from 0 to 43 mm and an overall mean of 25.64 ± 10.65 mm (Table 4). However, antibiotic responses varied markedly according to both the antimicrobial agent and the bacterial isolate tested. This variability was evident in the comparative distribution analysis, where antibiotics showed the widest range of inhibition zones, including values close to or equal to 0 mm (Figure 3). In comparison, CHH displayed a narrower inhibition range and a more homogeneous distribution than raw honey and the antibiotic panel, suggesting a more consistent antibacterial effect across the isolates tested, although with lower absolute inhibition than the most active antibiotics.

**TABLE 4:** Inhibition zone diameters of commercial antibiotics tested against clinical isolates of *Staphylococcus pseudintermedius*.

Antibiotic	Observations	Inhibition zone range (mm)	Mean ± SD (mm)
Amoxicillin–clavulanic acid	14	27–39	33.71 ± 5.00
Cefadroxil	14	25–37	31.86 ± 3.39
Cefpodoxime	14	21–35	30.57 ± 3.67
Cefovecin	14	37–43	39.14 ± 1.83
Chloramphenicol	14	9–37	23.79 ± 6.58
Minocycline	14	16–43	27.36 ± 7.46
Lincomycin	14	0–23	14.14 ± 7.47
Clindamycin	14	0–25	14.07 ± 11.55
Doxycycline	14	11–35	24.00 ± 7.80
Sulfamethoxazole–trimethoprim	14	0–27	17.79 ± 10.70

**FIGURE 3:**
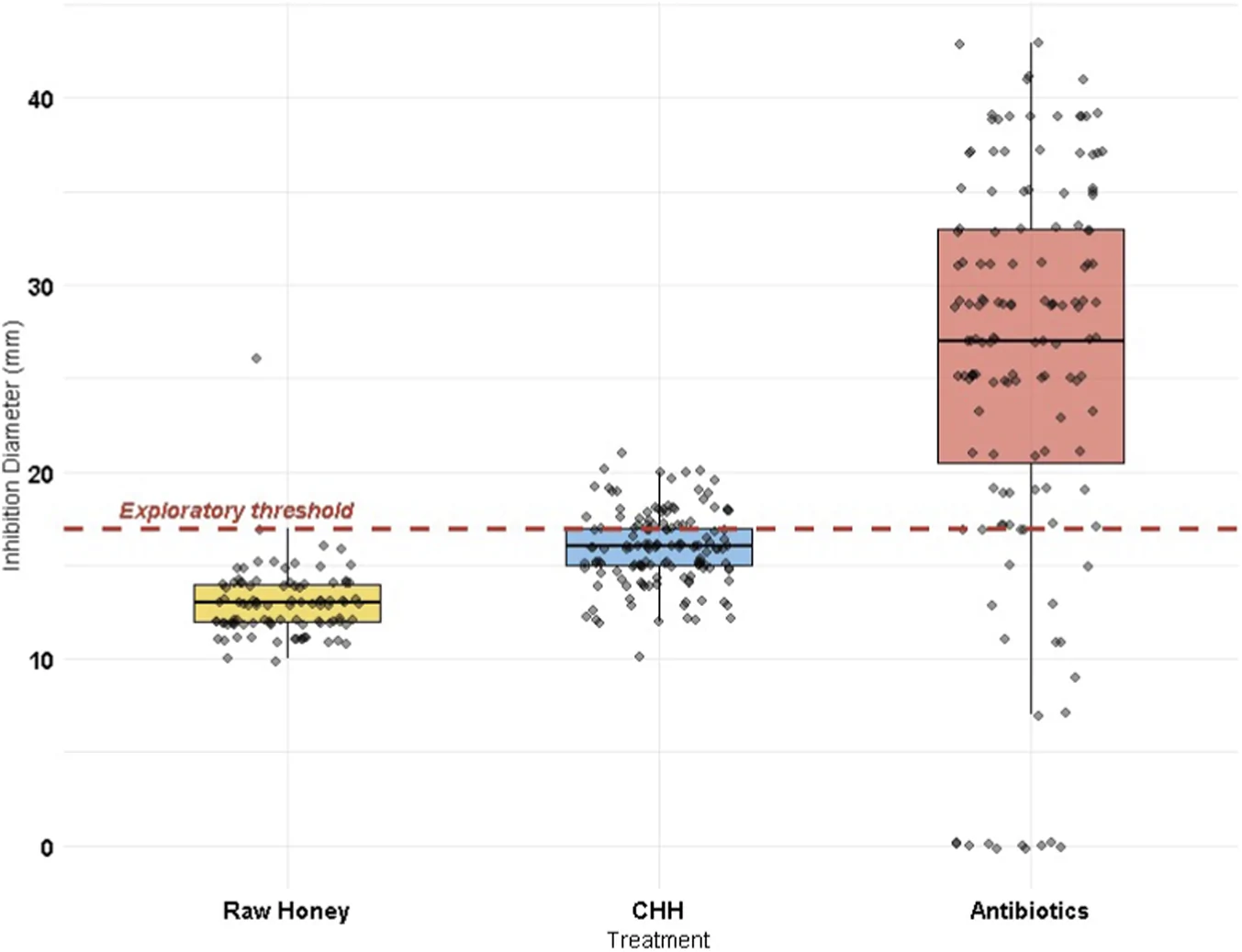
Comparative distribution of inhibition zone diameters obtained for raw honey, CHH formulations, and commercial antibiotics against clinical isolates of *Staphylococcus pseudintermedius*. Boxes represent the interquartile range, horizontal lines indicate the median, white diamonds indicate the mean, and gray dots represent individual observations. The dashed red line indicates the exploratory 17-mm reference threshold used only for comparative purposes.

An exploratory Pearson correlation analysis was performed to assess potential associations between honey physicochemical parameters and antibacterial activity, expressed as inhibition zone diameter (Figure 4). Because only eight honey samples were included, this analysis was considered hypothesis-generating. In raw honey, the strongest association was observed between antibacterial activity and HMF content (r = −0.88, *p* < 0.005). Other moderate associations, including those with °Brix and TPC, did not reach conventional statistical significance and should therefore be interpreted cautiously. In CHH formulations, moderate associations were observed for EC, pH, and °Brix; however, these associations were not statistically significant. Overall, these exploratory findings suggest that the physicochemical profile of honey may influence antibacterial activity, but they should not be interpreted as causal relationships or definitive predictors.

**FIGURE 4:**
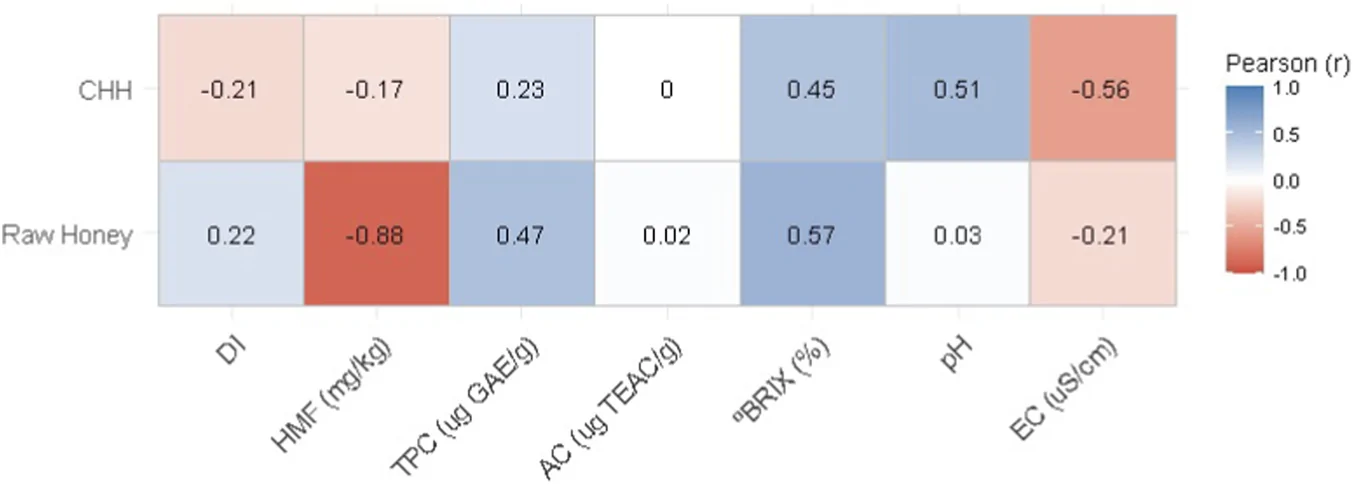
Exploratory Pearson correlation matrix between honey physicochemical parameters and antibacterial activity of raw honey and CHH formulations. Antibacterial activity was expressed as the inhibition zone diameter. Blue tones indicate positive correlations, red tones indicate negative correlations, and white indicates weak or no linear association. DI, diastase index; HMF, hydroxymethylfurfural; TPC, total phenolic content; AC, antioxidant capacity; °Brix, soluble solids; EC, electrical conductivity.

To improve transparency in the exploratory correlation analysis, the associations between HMF, TPC, and BRIX, and the inhibition zones of raw honeys and CHH, were also represented using scatter plots with individual honey samples and linear regression lines (Figure 5). These plots allow us to visualize the distribution of the eight honey samples and support the interpretation that the correlation results serve to generate hypotheses rather than to confirm them.

**FIGURE 5:**
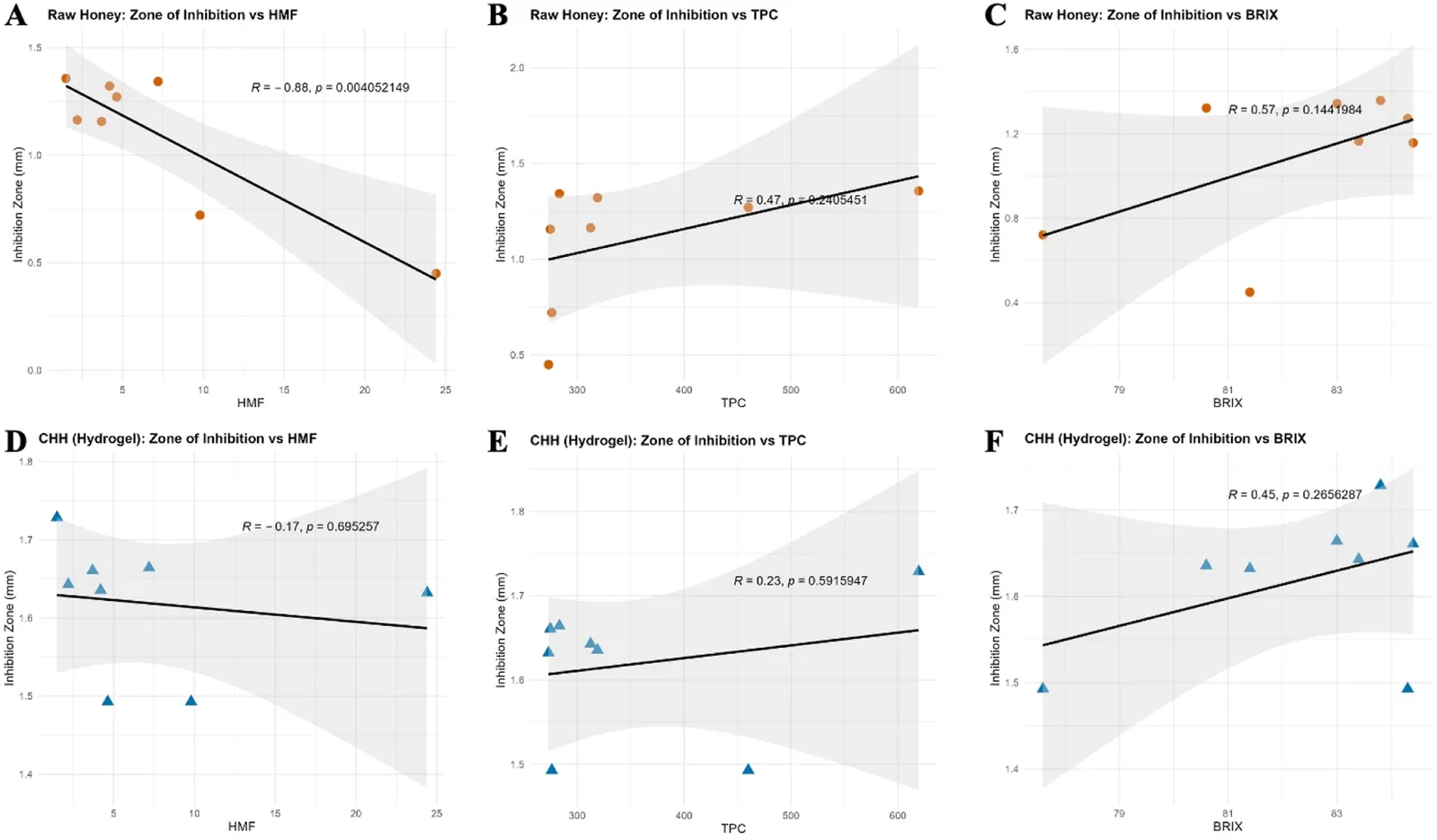
Exploratory associations between honey physicochemical parameters and antibacterial activity. **(A)** Scatter plot showing the association between HMF content and inhibition zone diameter for raw honey. **(B)** Scatter plot showing the association between TPC and inhibition zone diameter for raw honey. **(C)** Scatter plot showing the association between °Brix and inhibition zone diameter for raw honey. **(D)** Scatter plot showing the association between HMF content and inhibition zone diameter for CHH. **(E)** Scatter plot showing the association between TPC and inhibition zone diameter for CHH. **(F)** Scatter plot showing the association between °Brix and inhibition zone diameter for CHH. Dots represent individual honey samples or CHH, and lines indicate linear regression fits. Because only eight honey samples were included, these analyses should be interpreted as exploratory and hypothesis-generating. HMF, hydroxymethylfurfural; TPC, total phenolic content; °Brix.

The minimum inhibitory concentration assay further supported the antibacterial activity observed in the agar diffusion experiments (Table 5). Among the 14 clinical isolates of *Staphylococcus pseudintermedius*, raw honey samples showed higher and more variable MIC values, ranging from 7.81 to 500.00 mg/mL, with an overall mean value of 51.41 ± 90.68 mg/mL and a median MIC of 16.92 mg/mL. The overall MIC50 and MIC90 values for raw honey were 15.62 and 104.17 mg/mL, respectively. In contrast, CHH showed MIC values ranging from 3.12–16.67 mg/mL, with a mean value of 6.40 ± 3.83 mg/mL and a median MIC of 4.16 mg/mL. The MIC50 and MIC90 values were 4.16 and 10.42 mg/mL, respectively. A paired non-parametric comparison showed that CHH MIC values were lower than isolate-matched raw honey MIC values (Wilcoxon signed-rank test, *p* = 00,012), supporting the descriptive trend observed in the MIC distribution.

**TABLE 5:** MIC values of raw honey, CHH and selected antibiotics against clinical isolates of *Staphylococcus pseudintermedius*.

Treatment	Isolates	MIC range (mg/mL)	Mean ± SD (mg/mL)	Median MIC (mg/mL)	MIC50 (mg/mL)	MIC90 (mg/mL)
Raw honey	14	7.81–500	51.41 + 90.68	16.92	15.62	104.17
CHH	14	3.12–16.67	6.40 ± 3.83	4.16	4.16	10.42
Amoxicillin-clavulanic acid	14	8.00 × 10^-5^-2.08 × 10^−3^	4.64 × 10^−4^ ± 5.76 × 10^−4^	2.47 × 10^−4^	2.33 × 10^−4^	1.14 × 10^−3^
Cefadroxil	14	8.00 × 10^-5^-2.08 × 10^−3^	3.61 × 10^−4^ ± 6.48 × 10^−4^	1.07 × 10^−4^	1.07 × 10^−4^	1.67 × 10^−3^

MIC, values were calculated from triplicate determinations and used for descriptive analysis. MIC50 and MIC90 correspond to the lowest concentrations inhibiting at least 50% and 90% of the tested isolates or observations, respectively. Raw honey values include the eight honey samples tested against the 14 clinical isolates.

Amoxicillin–clavulanic acid showed MIC values ranging from 8.00 × 10^−5^ - 2.08 × 10^−3^ mg/mL, with a mean value of 4.64 × 10^−4^ ± 5.76 × 10^−4^ mg/mL and a median MIC of 2.47 × 10^−4^ mg/mL. The MIC50 and MIC90 values were 2.33 × 10^−4^ and 1.14 × 10^−3^ mg/mL, respectively. Cefadroxil showed MIC values ranging from 8.00 × 10^−5^ - 2.08 × 10^−3^ mg/mL, with a mean value of 3.61 × 10^−4^ ± 6.48 × 10^−4^ mg/mL and a median MIC of 1.07 × 10^−4^ mg/mL. The MIC50 and MIC90 values were 1.07 × 10^−4^ and 1.67 × 10^−3^ mg/mL, respectively.

Overall, CHH required lower concentrations to inhibit *S. pseudintermedius* growth than raw honey and showed a narrower MIC distribution across isolates. These findings are consistent with the agar diffusion results, where CHH produced larger and more homogeneous inhibition zones than raw honey. As expected, the selected antibiotics showed lower MIC values than CHH, confirming their stronger *in vitro* antibacterial potency under standardized microdilution conditions.

## 4 Discussion

This study provides an *in vitro* pharmacological evaluation of a Chilean honey-based hydrogel formulated with honey, chitosan, and gelatin for potential veterinary wound-related applications. The main findings indicate that CHH formulations were biocompatible with NIH/3T3 fibroblasts, increased cellular metabolic activity at higher honey-containing formulations, did not induce nitrite-mediated inflammatory activation, and showed improved antibacterial activity against clinical isolates of *S*. *pseudintermedius* compared with raw honey. Together, these results support further investigation of CHH as a candidate topical biomaterial, while highlighting that wound-healing efficacy was not assessed and requires functional and *in vivo* validation.

Before evaluating the biological effects of CHH, honey samples were characterized to verify their suitability as raw materials for hydrogel preparation. All samples met basic physicochemical quality criteria, including diastase index values above 8 Gothe units and HMF concentrations below 40 mg/kg, suggesting adequate enzymatic activity and limited thermal degradation or aging. These parameters are commonly used, together with pH, electrical conductivity, and soluble solids, to assess honey quality and processing conditions. In addition, the variability observed in color, antioxidant capacity, and total phenolic content is consistent with differences in floral origin and botanical contribution. This characterization is important because all natural products must specify basic parameters and their composition for standardization purposes. These parameters help identify and exclude altered or adulterated honeys and confirm that the composition of honey can influence both its biological activity and its performance once incorporated into a biomaterial matrix.

On the other hand, the antioxidant activity of the final hydrogel formulation was not evaluated, so future studies should evaluate antioxidant capacity, phenolic release, and redox-modulating effects directly in CHH formulations to determine whether incorporation into the chitosan–gelatin matrix preserves or modifies the antioxidant profile of Chilean honey.

### 4.1 *In vitro* effect of CHH on fibroblasts

Fibroblasts are essential for wound repair because they participate in extracellular matrix deposition, wound contraction, and tissue remodeling. In this study, CHH formulations did not reduce the metabolic activity of NIH/3T3 fibroblasts, suggesting that the hydrogel components were not cytotoxic under the experimental conditions tested. Moreover, CHH-50% and CHH-100% significantly increased fibroblast metabolic/reducing activity compared with untreated control cells. These findings are consistent with previous studies describing the favorable effects of honey and honey-containing biomaterials on wound-related cellular responses (Al-Jadi et al., 2014; Alvarez-Suarez et al., 2016; Yupanqui Mieles et al., 2022; Garabet et al., 2023). However, because the resazurin assay reflects cellular metabolic activity rather than direct cell proliferation, these results should be interpreted as evidence of biocompatibility and increased metabolic activity, rather than definitive proof of fibroblast proliferation.

The increase in resazurin reduction observed for CHH-50% and CHH-100% should be interpreted with caution. Resazurin reduction is commonly used as an indirect indicator of cell viability or metabolic activity, but it does not directly quantify cell number or proliferation (Kamiloglu et al., 2020). Honey-containing formulations may influence this assay through several mechanisms, including increased availability of carbohydrates such as glucose and fructose, changes in mitochondrial metabolic activity, modulation of cellular redox status, or possible contribution of reducing compounds to dye conversion. Therefore, although CHH did not reduce fibroblast metabolic activity and was compatible with NIH/3T3 cells under the tested conditions, the observed values above 100% should not be interpreted as definitive evidence of fibroblast proliferation. Future studies should include orthogonal assays such as direct cell counting, DNA quantification, Live/Dead staining, Ki-67 or BrdU/EdU incorporation, and migration assays to confirm whether CHH actively promotes fibroblast proliferation or wound-related cellular functions.

The enhanced metabolic response observed with CHH-50% and CHH-100% may be related not only to honey-derived bioactive compounds, such as carbohydrates, phenolics, flavonoids, amino acids, and micronutrients, but also to their incorporation into the gelatin–chitosan matrix. Gelatin can provide cell-interactive motifs that support fibroblast adhesion, while chitosan is widely used in wound-related biomaterials because of its biocompatibility and scaffold-forming properties (Binlateh et al., 2023; Garabet et al., 2023). Interestingly, the individual components and binary combinations did not significantly increase metabolic activity, whereas the complete CHH formulation did so at higher honey proportions. This suggests that the complete hydrogel structure may contribute to the observed cellular response. Nevertheless, additional assays, including direct cell counting, proliferation markers, collagen synthesis, and migration studies, are required to determine whether this increased metabolic activity translates into enhanced wound-healing capacity.

The use of NIH/3T3 murine fibroblasts represents a translational limitation of the present study. This cell line was selected as a standardized and reproducible preliminary model to evaluate cytocompatibility and wound-relevant cellular responses. However, NIH/3T3 fibroblasts do not fully reproduce the biological behavior of canine dermal fibroblasts, canine keratinocytes, resident immune cells, or the complex microenvironment of canine skin wounds. Therefore, the present results should be interpreted as preliminary evidence of *in vitro* cytocompatibility and nitrite response, rather than direct evidence of safety, efficacy, or wound-healing activity in dogs. Future studies should include canine dermal fibroblasts, canine keratinocytes, fibroblast–keratinocyte co-culture systems, *ex vivo* canine skin models, and *in vivo* wound-healing assays to better define the translational relevance of CHH for target veterinary patients.

### 4.2 Effect of CHH on nitrite production in murine fibroblasts

Nitric oxide is a signaling molecule involved in several stages of tissue repair, including inflammation, angiogenesis, extracellular matrix remodeling, and host defense. However, excessive or sustained nitric oxide production may contribute to oxidative stress and delayed wound resolution (Coleman, 2001; Durão et al., 2019; Zaborova et al., 2025). In this study, nitrite accumulation was used as an indirect indicator of nitric oxide production in NIH/3T3 fibroblasts. Raw honey significantly increased nitrite levels compared with the LPS-stimulated control, whereas none of the CHH formulations induced a significant increase in nitrite production.

The increase observed with raw honey may be related to its acidic pH, high osmolarity, and peroxide-generating activity, which can act as cellular stressors depending on concentration and exposure conditions. Honey has been reported to modulate wound-related cellular responses through mechanisms associated with acidity, hydrogen peroxide production, antioxidant compounds, and other bioactive molecules (Nolan et al., 2019; Tashkandi, 2021; Yupanqui Mieles et al., 2022). Interestingly, incorporation of honey into the gelatin–chitosan hydrogel matrix appeared to prevent the nitrite increase observed with raw honey alone. This may suggest that the polymeric network modulates the interaction between honey-derived compounds and fibroblasts, possibly through a more gradual exposure to honey components or through buffering/scavenging properties of the matrix components. Chitosan-based hydrogels have been widely investigated for wound applications because of their biocompatibility, scaffold-forming capacity, and ability to provide a hydrated microenvironment favorable for tissue repair (Elangwe et al., 2022; Şalva et al., 2023; Wang et al., 2023; Yasin et al., 2023).

The pH of CHH is another relevant translational factor that should be considered. Raw honey showed an acidic pH of approximately 3.0, whereas honey-containing formulations used in cell culture assays were adjusted to a final pH of approximately 5.0 to avoid excessive acidity-related effects on fibroblast viability. Although acidic conditions are part of the biological activity of honey and may contribute to antibacterial effects, pH can also influence cellular responses, redox balance, nitric oxide production, chitosan protonation, gelatin stability, and release behavior of honey-derived compounds. Therefore, the *in vitro* pH-adjusted conditions used in this study may not fully predict the behavior of CHH in a wound bed. *In vivo*, wound exudate, tissue buffering capacity, bacterial metabolism, inflammation, and healing stage may dynamically modify the local pH microenvironment. These changes could affect antioxidant and inflammatory responses, hydrogel degradation, release kinetics, and antibacterial activity. Future studies should evaluate CHH under simulated wound-fluid conditions, dynamic pH environments, and *in vivo* wound models to better determine how the wound microenvironment influences its biological and antibacterial performance.

Taken together, these findings suggest that CHH formulations increased fibroblast metabolic activity without inducing nitrite accumulation under the experimental conditions tested. However, this result should not be interpreted as evidence of anti-inflammatory activity or wound-healing efficacy, since only nitrite production was measured. Nitrite accumulation provides an indirect estimate of nitric oxide production but does not characterize the broader inflammatory or redox response induced by the formulation. In the present study, direct antioxidant markers, such as ROS production, superoxide dismutase activity, catalase activity, glutathione-related markers, or lipid peroxidation, were not evaluated. Similarly, inflammatory mediators such as iNOS expression, TNF-α, IL-1β, IL-6, IL-10, or other cytokines were not determined. Therefore, the antioxidant and anti-inflammatory pharmacological profile of CHH remains incomplete. Future studies should include oxidative stress assays, antioxidant enzyme activity, cytokine profiling, and canine dermal fibroblast or keratinocyte models to determine whether CHH actively modulates redox and inflammatory pathways relevant to tissue repair.

### 4.3 *In vitro* antibacterial effect of CHH against *Staphylococcus pseudintermedius*

Raw honey samples exhibited variable antibacterial activity against the clinical isolates of *S*. *pseudintermedius*, with inhibition zones ranging from 0 to 26 mm and a mean value of 10.98 ± 5.00 mm. This variability is expected for natural honey matrices, whose antibacterial activity may be influenced by botanical origin, physicochemical composition, viscosity, acidity, hydrogen peroxide generation, phenolic compounds, and other non-peroxide factors (Kwakman et al., 2010; Nolan et al., 2019; Tashkandi, 2021; Ogwu and Izah, 2025). In contrast, incorporation of honey into the gelatin–chitosan hydrogel increased the mean inhibition zone diameter to 16.19 ± 1.82 mm and produced a narrower distribution of responses. This suggests that the hydrogel matrix improved the antibacterial performance of honey under agar diffusion conditions, possibly by favoring a more homogeneous interaction between honey-derived bioactive compounds and the bacterial lawn. Similar advantages have been reported for honey-loaded hydrogel systems, which can improve handling, retention, and delivery of bioactive compounds in wound-related applications (Chopra et al., 2022; Yasin et al., 2023).

Because there are no validated clinical breakpoints for honey or honey-based hydrogels against *S. pseudintermedius*, the 17-mm value used in this study was interpreted only as an exploratory reference threshold. Using this threshold, 45 CHH observations reached or exceeded 17 mm, compared with only two observations for raw honey. Therefore, this shift should be interpreted as evidence of improved relative antibacterial activity of CHH compared with raw honey, rather than as a formal susceptibility classification. This distinction is important because interpretive criteria established for conventional antibiotics are antimicrobial and species-specific and cannot be directly extrapolated to complex natural matrices or biomaterial-based formulations (Papich, 2010; Humphries et al., 2020; Feßler et al., 2022).

The improved antibacterial activity observed for CHH compared with raw honey represents an experimental finding supported by both agar diffusion and MIC assays. However, the mechanisms underlying this effect were not directly investigated in the present study. Several hypotheses may explain this observation, including improved dispersion of honey-derived compounds within the test medium, matrix-associated delivery, stabilization of bioactive molecules, or an additional contribution from chitosan. Chitosan has been described as a polymer with antibacterial properties, mainly related to electrostatic interactions between its positively charged amino groups and negatively charged bacterial surfaces, which may alter membrane permeability (Elangwe et al., 2022). Nevertheless, the present study did not include release kinetics, diffusion assays, compound stability analyses, or combination studies designed to demonstrate synergistic interactions between honey and chitosan. Therefore, these mechanisms should be interpreted as plausible hypotheses rather than demonstrated explanations.

Compared with CHH, the commercial antibiotic panel produced larger inhibition zones overall, as expected for standardized antimicrobial agents. Nevertheless, antibiotic responses showed marked strain- and drug-dependent variability, including low or absent inhibition for some antibiotics. This finding is relevant because *S. pseudintermedius* is a major pathogen associated with canine pyoderma and has increasing clinical importance due to antimicrobial resistance, including methicillin-resistant and multidrug-resistant strains (Loeffler and Lloyd, 2018; Lynch and Helbig, 2021; Feßler et al., 2022). CHH presented lower absolute antibacterial potency than the most active antibiotics, but a more homogeneous response across isolates. These findings do not support CHH as a replacement for antibiotic therapy; rather, they suggest that honey-based hydrogels may represent a complementary topical strategy worthy of further investigation, particularly in the context of veterinary wound management and antimicrobial stewardship. To complement the agar diffusion results, preliminary MIC assays were performed for two clinically relevant antibiotics commonly used in canine pyoderma.

The MIC results further supported the antibacterial activity observed in the agar diffusion assays and provided a more quantitative comparison between raw honey, CHH, and selected antibiotics. As expected, amoxicillin–clavulanic acid and cefadroxil showed the lowest MIC values, confirming their strong *in vitro* activity against the clinical isolates of *S. pseudintermedius*. CHH also showed inhibitory activity, with lower and less variable MIC values than raw honey. The median MIC decreased from 16.92 for raw honey to 4.16 for CHH, while the MIC90 decreased from 104.17 to 10.42, respectively. These findings suggest that incorporation into the gelatin–chitosan matrix may improve the consistency and antibacterial performance of honey under broth microdilution conditions. However, because CHH remains less potent than standardized antibiotics, it should not be interpreted as a replacement for antimicrobial therapy. Rather, these results support further investigation of CHH as a complementary topical biomaterial for veterinary wound-related applications. Future studies should include time–kill assays, biofilm models, and *in vivo* wound models to better define the antibacterial potential and translational relevance of CHH.

### 4.4 Limitations of this study

Although the present findings support the *in vitro* biocompatibility and antibacterial activity of CHH, this work should be interpreted as a preliminary pharmacological evaluation rather than a complete characterization of a wound-healing biomaterial. The resazurin assay provides an indirect measure of cellular metabolic/reducing activity and does not demonstrate fibroblast proliferation, migration, collagen synthesis, or extracellular matrix deposition. Likewise, nitrite quantification provides only an indirect indicator of nitric oxide-mediated activation and does not fully characterize inflammatory, antioxidant, or oxidative stress pathways. Therefore, the present findings should be restricted to the measured *in vitro* outcomes and should not be interpreted as evidence that CHH accelerates or improves wound healing.

Another important limitation is the absence of a detailed physicochemical, structural, and chemical characterization of the final hydrogel formulation. Although raw honey samples were characterized for quality parameters, antioxidant capacity, and total phenolic content, the final chitosan–gelatin matrix was not assessed for swelling, degradation, rheological behavior, porosity, morphology, mechanical properties, release kinetics, or post-formulation active markers. Consequently, the improved antibacterial activity observed for CHH compared with raw honey cannot be definitively attributed to hydrogel architecture, controlled release, enhanced diffusion, stabilization of honey-derived compounds, or additive/synergistic interactions with chitosan. A comprehensive characterization of CHH is currently being developed as part of a separate formulation-focused study.

The use of NIH/3T3 murine fibroblasts also limits the direct extrapolation of the cellular findings to canine skin wounds. Although this cell line is useful for preliminary cytocompatibility screening, canine dermal fibroblasts, keratinocytes, immune-cell interactions, and the wound microenvironment may respond differently to CHH. Future studies should include canine skin cell models, co-culture systems, *ex vivo* canine skin, direct antioxidant and inflammatory markers, bactericidal assays, time–kill kinetics, antibiofilm models, and *in vivo* wound-healing assays to define the translational relevance of CHH for veterinary patients.

## 5 Conclusion

This study provides preliminary *in vitro* pharmacological evidence supporting the biocompatibility and antibacterial activity of a Chilean honey hydrogel (CHH) formulated with honey, chitosan, and gelatin for potential veterinary wound-related applications. Under the experimental conditions tested, CHH formulations did not reduce NIH/3T3 fibroblast metabolic activity, increased resazurin reduction at higher honey-containing proportions, and did not increase nitrite production. However, direct antioxidant and inflammatory markers were not evaluated.

CHH also improved the antibacterial activity of raw honey against clinical isolates of *Staphylococcus pseudintermedius*, producing larger and more homogeneous inhibition zones and lower, less variable MIC values than raw honey. Nevertheless, the mechanisms underlying this effect remain to be determined. They cannot yet be attributed specifically to hydrogel architecture, controlled release, enhanced diffusion, stabilization of honey-derived compounds, or additive/synergistic interactions with chitosan.

Overall, these findings support further investigation of CHH as a candidate topical biomaterial for veterinary wound-related applications. However, the present results should be interpreted strictly as preliminary *in vitro* evidence and do not demonstrate wound-healing efficacy. Future studies should include post-formulation chemical and structural characterization, active-marker quantification, release kinetics, stability assays, evaluation under simulated wound-fluid and dynamic pH conditions, fibroblast migration, collagen synthesis, extracellular matrix deposition, Live/Dead and apoptosis assays, ROS and cytokine profiling, bactericidal and antibiofilm assays, canine skin cell models, and *in vivo* wound-healing studies to define the therapeutic relevance and translational potential of CHH for veterinary wound management.

## Data Availability

The raw data supporting the conclusions of this article will be made available by the authors, without undue reservation.
